# Analysis of a silicon comb structure using an inverse Talbot–Lau neutron grating interferometer

**DOI:** 10.1038/s41598-022-06409-y

**Published:** 2022-03-03

**Authors:** Youngju Kim, Daeseung Kim, Daniel S. Hussey, Jongyul Kim, Mona Mirzaei, Dmitry A. Pushin, Charles W. Clark, Seung Wook Lee

**Affiliations:** 1 School of Mechanical Engineering, Pusan National University, Busan, Republic of Korea; 2 Department of Chemistry and Biochemistry, University of Maryland, College Park, MD USA; 3 Neutron Physics Group, National Institute of Standards and Technology, Gaithersburg, MD USA; 4 Neutron Science Division, Korea Atomic Energy Research Institute, Daejeon, Republic of Korea; 5 Institute of Quantum Computing, University of Waterloo, Waterloo, ON Canada; 6 Department of Physics and Astronomy, University of Waterloo, Waterloo, ON Canada; 7 Science Systems and Applications, Inc., Lanham, MD USA; 8 Joint Quantum Institute, National Institute of Standards and Technology and the University of Maryland, Gaithersburg, MD USA

**Keywords:** Engineering, Techniques and instrumentation, Optics and photonics

## Abstract

We describe an inverse Talbot–Lau neutron grating interferometer that provides an extended autocorrelation length range for quantitative dark-field imaging. To our knowledge, this is the first report of a Talbot–Lau neutron grating interferometer (nTLI) with inverse geometry. We demonstrate a range of autocorrelation lengths (ACL) starting at low tens of nanometers, which is significantly extended compared to the ranges of conventional and symmetric setups. ACLs from a minimum of 44 nm to the maximum of 3.5 μm were presented for the designed wavelength of 4.4 Å in experiments. Additionally, the inverse nTLI has neutron-absorbing gratings with an optically thick gadolinium oxysulfide (Gadox) structure, allowing it to provide a visibility of up to 52% while maintaining a large field of view of approximately 100 mm × 100 mm. We demonstrate the application of our interferometer to quantitative dark-field imaging by using diluted polystyrene particles in an aqueous solution and silicon comb structures. We obtain quantitative structural information of the sphere size and concentration of diluted polystyrene particles and the period, height, and duty cycle of the silicon comb structures. The optically thick Gadox structure of the analyzer grating also provides improved characteristics for the correction of incoherent neutron scattering in an aqueous solution compared to the symmetric nTLI.

## Introduction

Neutron grating interferometry^1–5^ is a novel imaging technique that provides structural information regarding materials based on dark-field contrast, which is related to small-angle neutron scattering (SANS). The dark-field contrast for a given ACL can be used to derive the projected real-space correlation function of the microstructure of materials^6–9^. The projected real-space correlation function is related to the autocorrelation of the density distribution of a structure, which can be used to extract the shape and order of a structure based on the relationship defined by the Abel transform^10–12^.

In dark-field imaging, the ACL is a system parameter of a nTLI that is related to the wavelength of the neutron beam, period of the analyzer grating, and distance between the sample and analyzer grating^9^. Dark-field imaging using a conventional nTLI^13,14^ has an ACL of several micrometers, meaning it can only analyze sample inhomogeneities in the micrometer range, unlike the conventional range of SANS which is of order nanometers. Therefore, different geometries are required to extend the ACL range to apply dark-field imaging to material studies that have traditionally been conducted using conventional SANS instruments.

In this regard, a nTLI with the conventional geometry using a high fractional Talbot distance instead of the first fractional Talbot distance can be used for quantitative dark-field imaging^15,16^. This makes it possible to realize a smaller ACL down to hundreds of nanometers than possible in the first Talbot-distance by positioning a sample closer to the analyzer grating and increasing the distance between the phase grating and analyzer grating.

A far-field grating interferometer^17–20^ and single absorbing grating^21^ that can alter ACL based on the period of the moiré pattern have been studied by directly reading the pattern using such detectors without using analyzer gratings, which achieves an ACL range from tens of nanometers to several micrometers.

The geometrical arrangement of nTLI results in the variation of the grating configuration^22^, which allow to optimize the measurement configuration to explore different aspect of the sample. To investigate a broad range of ACL in dark-field imaging, in particular to be sensitive to small ACLs, the symmetric and inverse geometries rather than the conventional nTLI are advantage because of the larger period of analyzer grating. It has been demonstrated that the symmetric nTLI^23^ that has the potential for extending the ACL to tens of nanometers by using an analyzer grating with a large period. However, an inverse nTLI has the largest period of analyzer grating among the three geometries of nTLIs for the same total system length, thus the inverse nTLI can extend the ACL even further.

In this paper, we present the application of an inverse nTLI to quantitative dark-field imaging. Our setup represents the first report of a nTLI with the inverse geometry. It can achieve the continuous measurement of dark-field contrast values at nanometer-scale ACL for accurate structural analysis. Inverse nTLI also provides significant improvements in visibility and correction of incoherent neutron scattering over systems based on the optically thick neutron-absorbing structures of source and analyzer gratings. We assess the quantitative accuracy of our dark-field imaging setup by analyzing a well-known reference sample system of diluted polystyrene particles in an aqueous solution, as well as three different silicon comb structures. We show that the inverse geometry yields reasonable results for the structural information of each system and compare interferometer performance with symmetric nTLI.

We use SI units throughout, but in keeping with convention in the neutron scattering community, neutron wavelengths are reported in Ångstrom units (1 Å = 0.1 nm).

## Experimental setup for the inverse Talbot–Lau neutron grating interferometer

**Figure 1:**
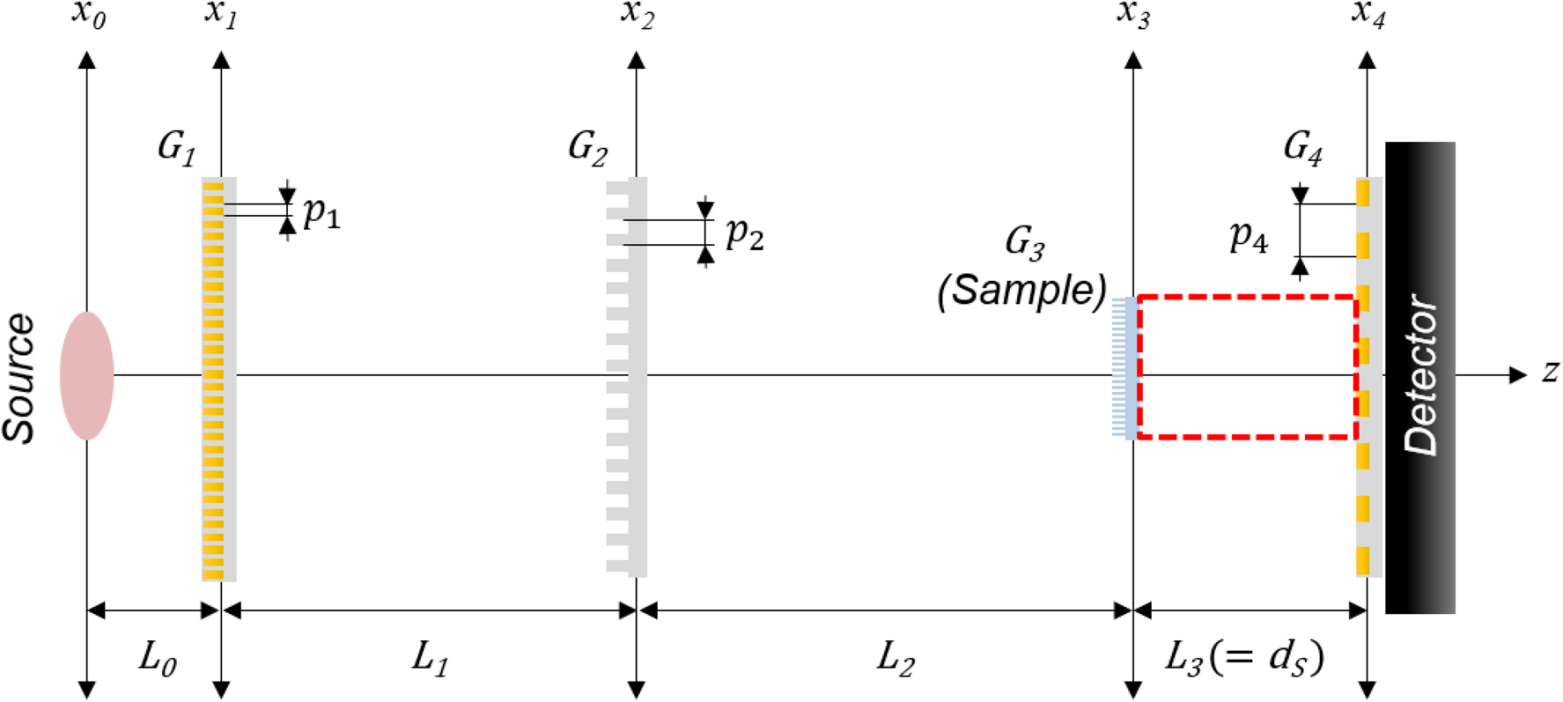
Schematic of an inverse Talbot–Lau neutron grating interferometer consisting of a source grating (G_1_), phase grating (G_2_), and analyzer grating (G_4_). The inter-grating distance L_1_ is less than L_2_ + L_3_ and this setup uses the first fractional Talbot order. The sample (diluted polystyrene particle solution or G_3_ for silicon comb structure) is placed between G_2_ and G_4_, and moves along the ${\varvec{z}}$-axis to vary the ACL of dark-field imaging. The red box is the detection area of the projection of G_3_ in the interference pattern simulation presented in Supplementary Materials.

Figure 1 shows the schematic of an inverse nTLI. Experiments were conducted at the cold neutron imaging beamline NG6 at the NIST Center for Neutron Research^24^. The nTLI was an inverse-type interferometer of the first fractional Talbot order. The inverse nTLI was designed so that the total system length is almost that of the total length of the imaging beamline, while empolying the same phase grating as the symmetric nTLI in Table 1. The inverse nTLI has characteristics of gratings and inter-grating distances which are exactly opposite to those of the conventional nTLI, that is, the largest period of analyzer grating, the smallest period of source grating, and the shortest distance between source grating and phase grating. Additional details are provided in Table 1 and a full description is provided below.

**Table 1:** Detailed parameters of inverse and symmetric Talbot–Lau neutron grating interferometers.

Type		Inverse	Symmetric
Designed wavelength	$\lambda$	4.4 Å	4.4 Å
Fractional Talbot order	$m$	1	1
Inter-grating distance	G_1_ − G_2_ (L_1_)	3030 mm	3636 mm
	G_2_ − G_4_ (L_2_ + L_3_)	4545 mm	3636 mm
Period of gratings	p_1_	67 μm	80 μm
	p_2_	80 μm	80 μm
	p_4_	100 μm	80 μm
Height of gratings	h_1_	150 μm (Gadox)	100 μm (Gadox)
	h_2_	34.39 μm (Silicon)	34.39 μm (Silicon)
	h_4_	150 μm (Gadox)	100 μm (Gadox)
Duty cycle of gratings	d_1_	0.75 (Gadox)	0.75 (Gadox)
	d_2_	0.5 (Silicon)	0.5 (Silicon)
	d_4_	0.5 (Gadox)	0.5 (Gadox)

The source grating (G_1_) had a one-dimensional line structure consisting of Gadox (Gd_2_O_2_S) with an opening ratio of 0.25 and period of 67 μm. The analyzer grating (G_4_) had a one-dimensional line structure consisting of Gadox with an opening ratio of 0.5 and period of 100 μm. For both G_1_ and G_4_, the height of the Gadox structure was 150 μm. The Gadox structures were fabricated using the Gadox powder filling method^25^. The phase grating (G_2_) had a one-dimensional line structure etched in a silicon wafer with an opening ratio of 0.5 and period of 80 μm. The height of the silicon structure was 34.39 μm, resulting in a π-phase shift for 4.4 Å neutrons. This structure was fabricated using the silicon deep wet etching method in KOH solution.

The inverse nTLI used a monochromatic 4.4 Å neutron beam produced by a double-crystal monochromator (DCM). The DCM was installed between G_1_ and a circular beam aperture with a radius of 13 mm. The detector was an Andor sCMOS camera with a 50 mm lens, resulting in a 2560 × 2160 pixels area with an effective pixel pitch of 51.35 μm. The scintillator was a LiF:ZnS screen of 300 μm in thickness. The detector was installed immediately after G_4_.

Dark-field images of samples were processed using Fourier analysis based on twelve phase step images obtained via the phase stepping method for G_2_^26^. Three exposures were merged using a median filter for each phase step image. The exposure times were 100 s and 50 s for the diluted polystyrene particles in an aqueous solution and the silicon comb structures, respectively, as described below.

### Samples

Quantitative dark-field imaging using the inverse nTLI was conducted using diluted polystyrene particles in an aqueous solution and comb structures etched in silicon. The polystyrene particles had diameters of 0.15 μm, 0.31 μm, 0.6 μm, and 1 μm, and were diluted in an aqueous solution of H_2_O (volume fraction of 59%) and D_2_O (volume fraction of 41%). The concentration of polystyrene particles was 6% by volume for all solutions and the solutions were sealed in quartz cuvettes with a thickness of 5 mm. The polystyrene particles in aqueous solutions represented structural systems of monodisperse particles based on the density of the solutions (H_2_O density of 1.0 g/cm^3^ and D_2_O density of 1.11 g/cm^3^), which was approximately equal to that of polystyrene (polystyrene density of 1.05 g/cm^3^). The difference between the neutron scattering length densities of the polystyrene particles and the solution at a neutron wavelength of 4.4 Å was $\Delta {\rho }_{0}$ = 87.8 μm^−2^.

Additionally, quantitative dark-field imaging was conducted for different D_2_O concentrations (and different H_2_O concentrations) to evaluate the incoherent scattering correction of the pure aqueous solution, which was essential for dark-field imaging of the diluted polystyrene particle solutions. The pure aqueous solutions were composed of D_2_O and H_2_O. The solutions were sealed in quartz cuvettes with thicknesses of 5 mm with 0%, 43%, 57%, 71%, and 100% by volume of D_2_O.

The silicon comb structure was a binary Ronchi grating with an opening ratio of 0.5 and three different structures were tested in our experiments. The silicon comb structures are noted as the first, second, and third structures in the manuscript. The details of silicon comb structures of periods, heights, and duty cycles are provided in the nominal section of Table 3. All silicon comb structures were fabricated using cryogenic and Bosch processes^27,28^ and had a fabricated area of 20 mm × 20 mm on a 102 mm diameter silicon wafer. For a silicon density of 2.32 g/cm^3^, the difference between the neutron scattering length densities of silicon and air at a neutron wavelength of 4.4 Å is $\Delta {\rho }_{0}$ = 206.5 μm^−2^.

**Figure 2:**
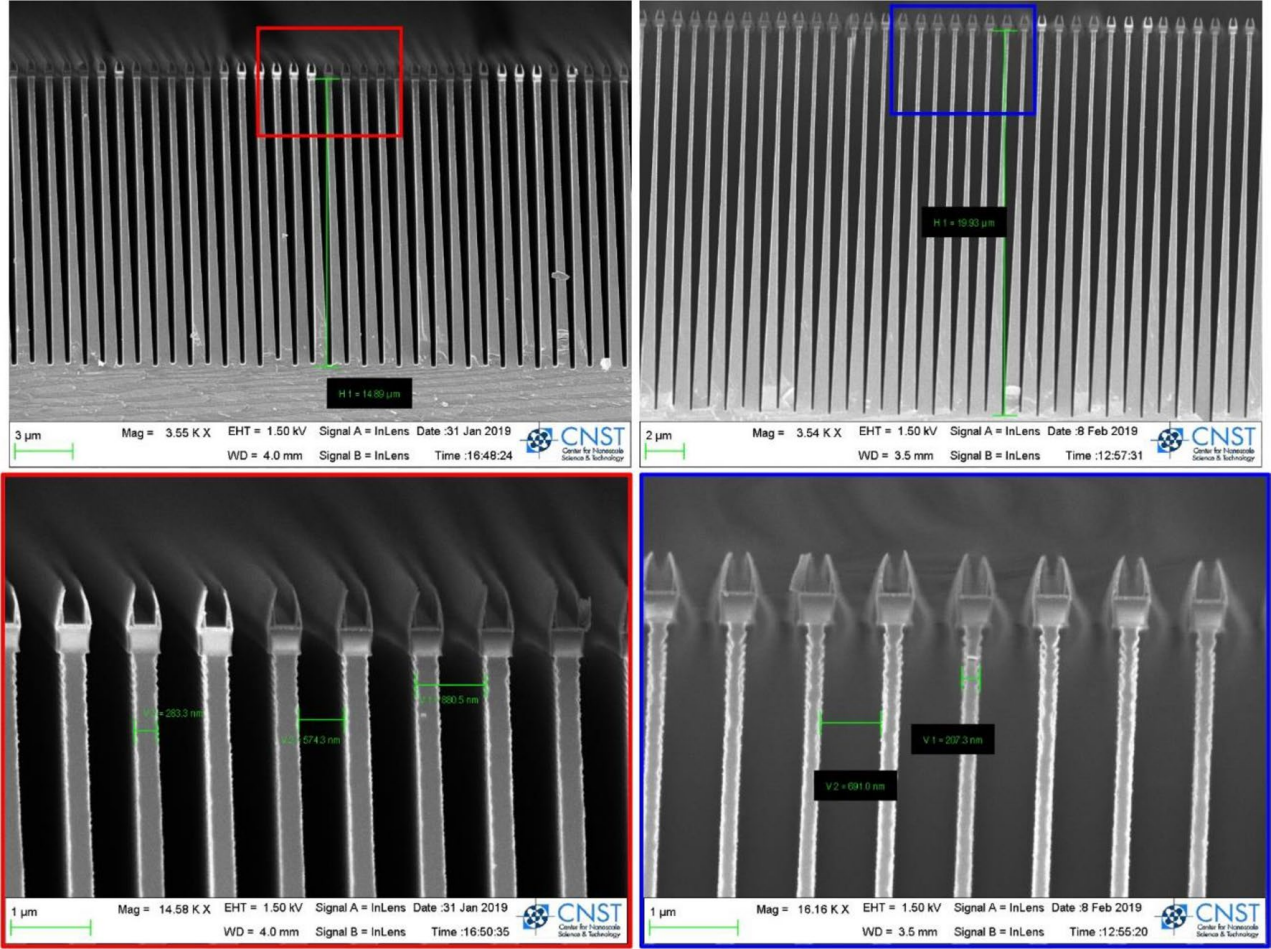
The cross-section SEM images of the silicon comb structure. Both structures have the duty cycle of 0.5 and period of 0.9 μm, and each height is 15 μm (left) and 20 μm (right) corresponding the first and second structures. The red and blue boxes show the detailed view of upper bars of each structure. The mask on the top of bar was removed before the experiment.

Figure 2 shows the cross-section scanning electron microscopic (SEM) images of the first and second silicon comb structures. For the second structure, it is difficult to determine the duty cycle due to the triangular shape of the structure, but it is reported to be 0.5 through the nearly equal spacing of bars and openings.

### Data analysis

The small angle scattering originating from a material reduces the visibility, that is the amplitude of the interference pattern, and the ratio of the visibility of the modulation induced with a sample (${V}_{s}$) to that without a sample (${V}_{o}$), provides the dark-field contrast. The dark-field contrast is expressed as a function of the ACL, $\xi ,$ as^9^

1 $$DFI\left(\xi \right)={V}_{s}/{V}_{o}={e}^{{\Sigma }_{s}t\left(G\left(\xi \right)-1\right)} ,$$

where ${\Sigma }_{s}$ is the total neutron scattering cross section, $t$ is the thickness of the sample, and $G\left(\xi \right)$ is the projected real-space correlation function.

The ACL, $\xi ,$ is a system parameter of the grating interferometer, which is defined by^7^

2 $$\xi =\lambda {d}_{s}/p_{m} ,$$

where $\lambda$ is the wavelength of the neutron beam, ${d}_{s}$ is the distance between the sample and the analyzer grating, and $p_{m}$ is the period of the moiré pattern. In a nTLI, $p_{m}$ is equal to the analyzer grating period p_2_.

The ACL represents the size at which correlations are measured. Therefore, the shorter the ACL of the grating interferometer, the smaller the material structures that can be analyzed. The inverse nTLI has a larger analyzer grating period compared to the conventional and symmetric nTLIs, resulting in the smallest ACL among the nTLI geometeries.

In our experiments, dark-field contrast was measured over a range of ACLs by varying the distance between the sample and the analyzer grating. The ACL ranged from 44 nm to 3.5 μm for sample distances ranging from 10 mm to 790 mm from the analyzer grating with a neutron wavelength of 4.4 Å and an analyzer grating period of 100 μm.

### Diluted polystyrene particle solution (isolated sphere model)

Values of ${\Sigma }_{s}$ and $G\left(\xi \right)$ are determined by the material and its scattering structure. For diluted polystyrene particles in an aqueous solution, the scattering structure is monodisperse particles with a low concentration, which is described by the isolated sphere model. The projected real-space correlation function of the isolated particle model is approximated as^11^

3 $$G\left(\xi \right)={e}^{-\frac{9}{8}{\left(\frac{\xi }{r}\right)}^{2}} ,$$

where $r$ is the sphere radius.

The total neutron scattering cross section of the isolated particle model is given by^13^

4 $${\Sigma }_{s}=\frac{3}{2}{\lambda }^{2}{\Delta {\rho }_{0}}^{2}{\varphi }_{V}r ,$$

where ${\varphi }_{V}$ is the volume fraction (i.e., concentration of particles).

### Silicon comb structure (oriented periodic bars)

**Figure 3:**
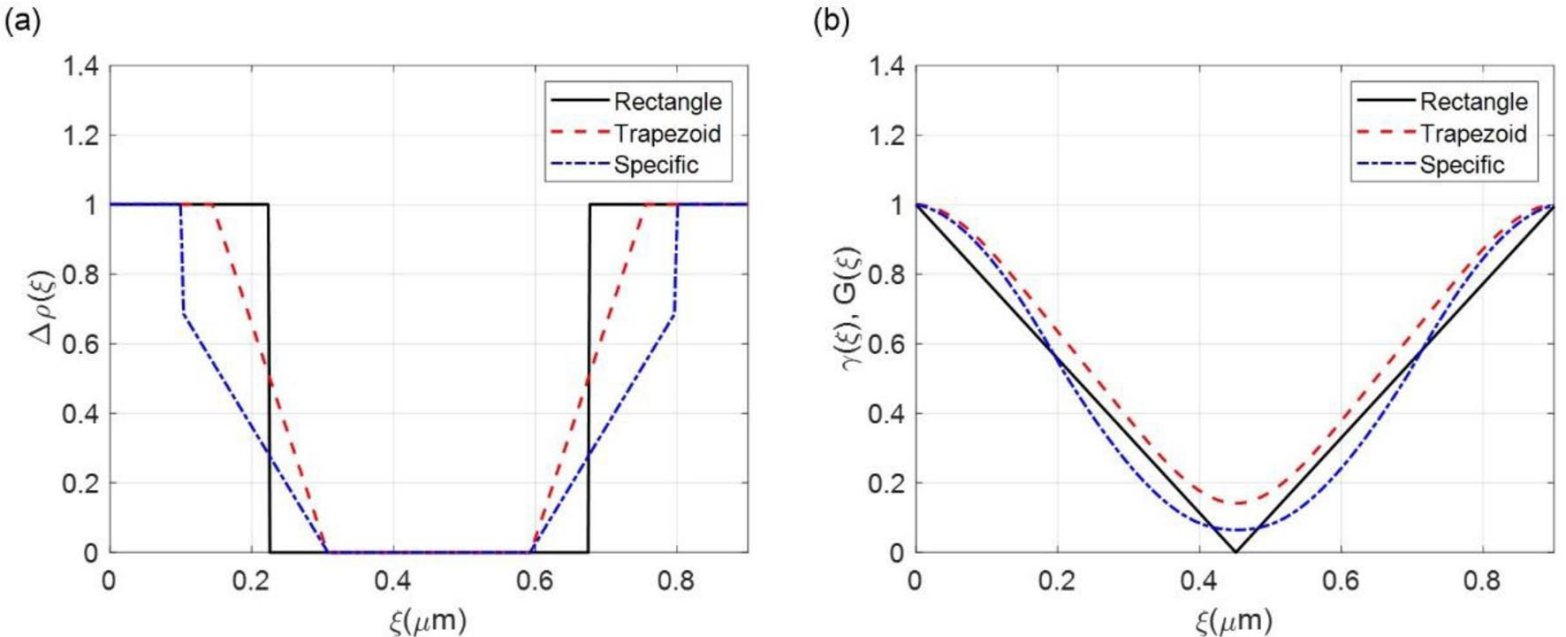
(**a**) The density distribution, and (**b**) autocorrelation and projected real-space correlation functions of the oriented periodic bars for the silicon comb structure. The period of the silicon comb structure is 0.9 μm. The shapes are rectangle (black solid line), trapezoid (red dashed line), and specific shape (blue dash-dotted line). The specific shape has a rectangular top and a trapezoidal bottom.

For the silicon comb structure (G_3_), which is the binary Ronchi grating, the scattering structure is described by oriented periodic bars of rectangular structure (black solid line) as shown in Fig. 3(a). In this case, the density distribution can be given as^29,30^

5 $$\rho \left(\mathbf{r}\right)=\rho \left(x,z\right)=\sum_{a=-\infty }^{\infty }\Pi \left(x,{d}_{x}\right)\Pi \left(z,{d}_{z}\right)f\left(x,p\right),$$

with

6 $$\Pi \left(x,{d}_{x}\right)=\left\{\begin{array}{cc}1,& \left|x-ap\right|\le {d}_{x}/2\\ 0,& \left|x-ap\right|>{d}_{x}/2\end{array}\right. ,$$

where $f\left(x,p\right)=\mathrm{sgn}\left(\mathrm{cos}\left(2\pi x/p\right)\right),$ is the square wave function, $p$ is the period of the bars, $a$ is the integer, and ${d}_{x}$ and ${d}_{z}$ are the lengths of the bars in the $x$- and $z$-directions, respectively and the grating lines are parallel to the $y$-axis. As shown in Fig. 3, the autocorrelation function $\gamma \left(\xi \right)$ of such a distribution is a triangular function. When calculated across the bars, the projected real-space correlation function $G\left(\xi \right)$ is also a triangular function because $x$ and $z$ can be separated in the nTLI^11^. The projected real-space correlation function $G\left(\xi \right)$ can be expressed as

7 $$G\left(\xi \right)=\sum_{a=-\infty }^{\infty }\Lambda \left(\xi ,{d}_{x}\right)f\left(\xi ,p\right),$$

with

8 $$\Lambda \left(\xi ,{d}_{x}\right)=\left\{\begin{array}{cc}1-\frac{1}{{d}_{x}}\left|\xi \right|,& \left|\xi -ap\right|\le {d}_{x}\\ 0,& \left|\xi -ap\right|>{d}_{x}\end{array}\right. .$$

However, the actual silicon comb structures are not perfectly oriented periodic bars in a rectangular shape. As shown in Fig. 2, the comb structure is somewhat trapezoidal, with a narrower width at the top of the bar than at the bottom, due to undercuts and scallops that occur during the etching process. As the height of the structure increases, the upper bar becomes very narrow, and the lower portion becomes wider. We refer to our model of the structure as “the specific shape”. Figure 3(a) and (b) respectively show the density distribution and its autocorrelation function for the ideal silicon comb structures of the rectangular shapes (black solid line) and the actual silicon comb structures of the trapezoidal (red dashed line) and specific (blue dotted-dash line) shapes.

The density distribution shown in Fig. 3(a) is predicted through the SEM images in Fig. 2. The real-space correlation function approximates the autocorrelation function, and the two functions are equivalent for the oriented periodic bars in a rectangular shape. The opening ratio of upper bar is 0.68 and the opening ratio of lower bar is 0.32 for the trapezoidal shape. The opening ratio of upper bar is 0.78 and the opening ratio of lower bar is 0.32 for the specific shape. In the specific shape, the height of rectangular upper bar is a ratio of 0.3 to the total bar height. For the silicon comb structures, the trapezoidal shape represents the first and third structure, and the specific shape represents the second structure in Table 2.

**Table 2:** Structural parameters of diluted polystyrene particle solutions based on the isolated sphere model.

Nominal		Experimental (fitted)	
Diameter (μm)	Concentration	Diameter (μm)	Concentration
0.15	0.06	0.11 ± 0.03	0.07 ± 0.04
0.31	0.06	0.36 ± 0.07	0.03 ± 0.01
0.6	0.06	0.52 ± 0.03	0.05 ± 0.01
1	0.06	0.98 ± 0.04	0.05 ± 0.01

Note that errors represent confidence bounds on parameters of the confidence level of 95%.

The total neutron scattering cross section and thickness (i.e., bar height) is defined as^31^

9 $${\Sigma }_{s}t=\left(n{d}_{x}{d}_{y}{d}_{z}\right){\left(\Delta {\rho }_{0}\lambda {d}_{z}\right)}^{2}$$

where ${d}_{x}$ is the bar width, ${d}_{y}$ is the bar length, and ${d}_{z}$ is the bar height parallel to the incident neutron beam. Additionally, $n$ is the bar density, which is the number of bars over the volume. The term $n{d}_{x}{d}_{y}{d}_{z}$ refers to the duty cycle of the structure, which is defined as the ratio of the bar width to the period, and the term is assumed to be equivalent to all cases of silicon comb structures.

## Experimental results and discussion

### Improved visibility

**Figure 4:**
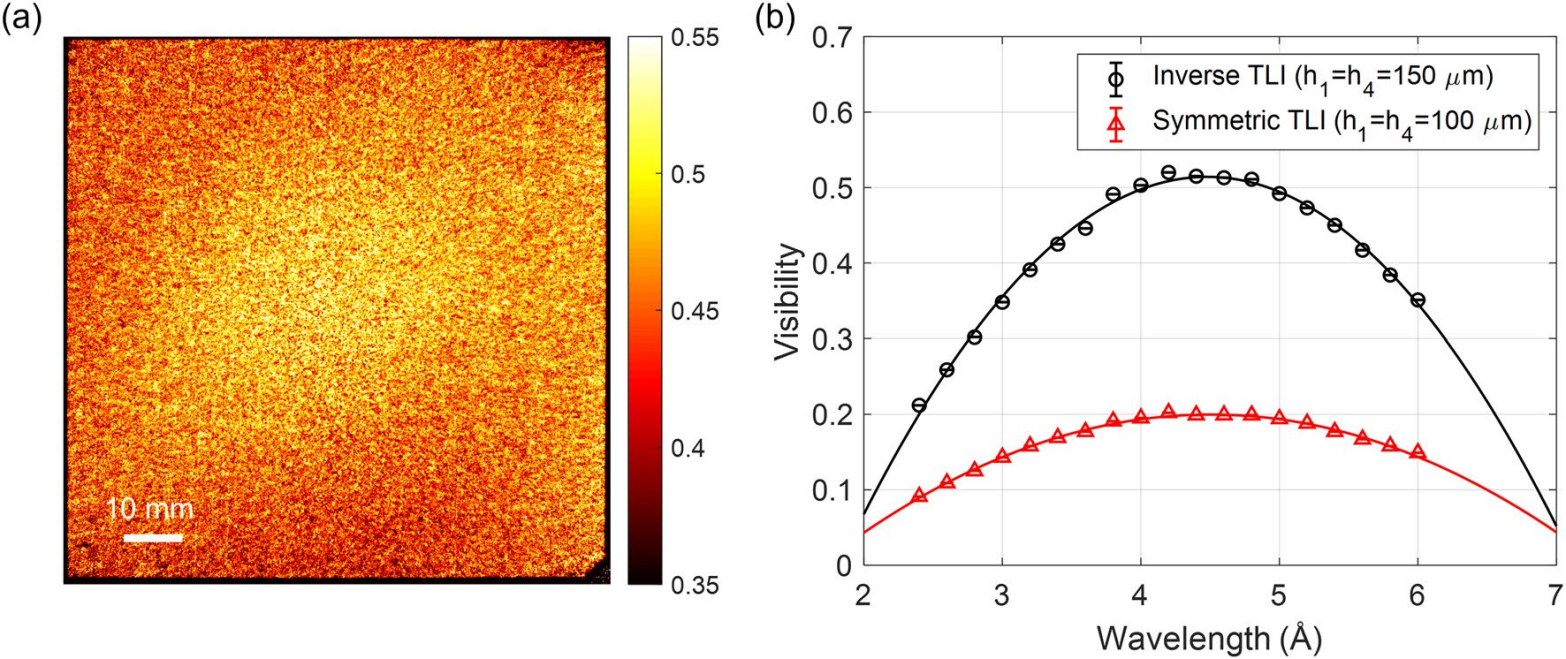
(**a**) Visibility map of the inverse Talbot–Lau neutron grating interferometer at a wavelength of 4.4 Å. (**b**) Visibility results for inverse and symmetric setups according to wavelength. The wavelength was varied from 2.4 Å to 6 Å using a DCM. The inverse setup uses a height of 150 μm for source and analyzer gratings, which improves visibility compared to the symmetric setup (100 μm height for source and analyzer gratings).

Figure 4 presents the visibility of the inverse and symmetric nTLIs shown in Table 1. A visibility map of inverse nTLI measured at a neutron wavelength of 4.4 Å is presented in Fig. 4(a) and the wavelength dependence of the visibility is presented in Fig. 4(b). The visibility map of the inverse geometry reveals a reasonably uniform distribution of visibility over the entire fabricated area of approximately 100 mm × 100 mm. The wavelength was varied from 2.4 Å to 6 Å. The region of interest (ROI) for visibility was set to 700 × 700 pixels and the standard errors of each ROI are represented by the error bars in Fig. 4(b). The average visibility at 4.4 Å is 51%. The measured maximum visibility is 52% at 4.2 Å and the maximum visibility based on parabolic interpolation is expected to be 52% at 4.5 Å, which shows the system is well developed. The inverse setup significantly improves the visibility compared to the symmetric setup detailed in Table 1. The height of the absorption gratings (G_1_ and G_4_) in the inverse setup is 150 μm, which is greater than the absorption grating height of 100 μm in the symmetric setup, resulting in enhanced neutron absorption and visibility.

### Correction of incoherent scattering from aqueous solution

**Figure 5:**
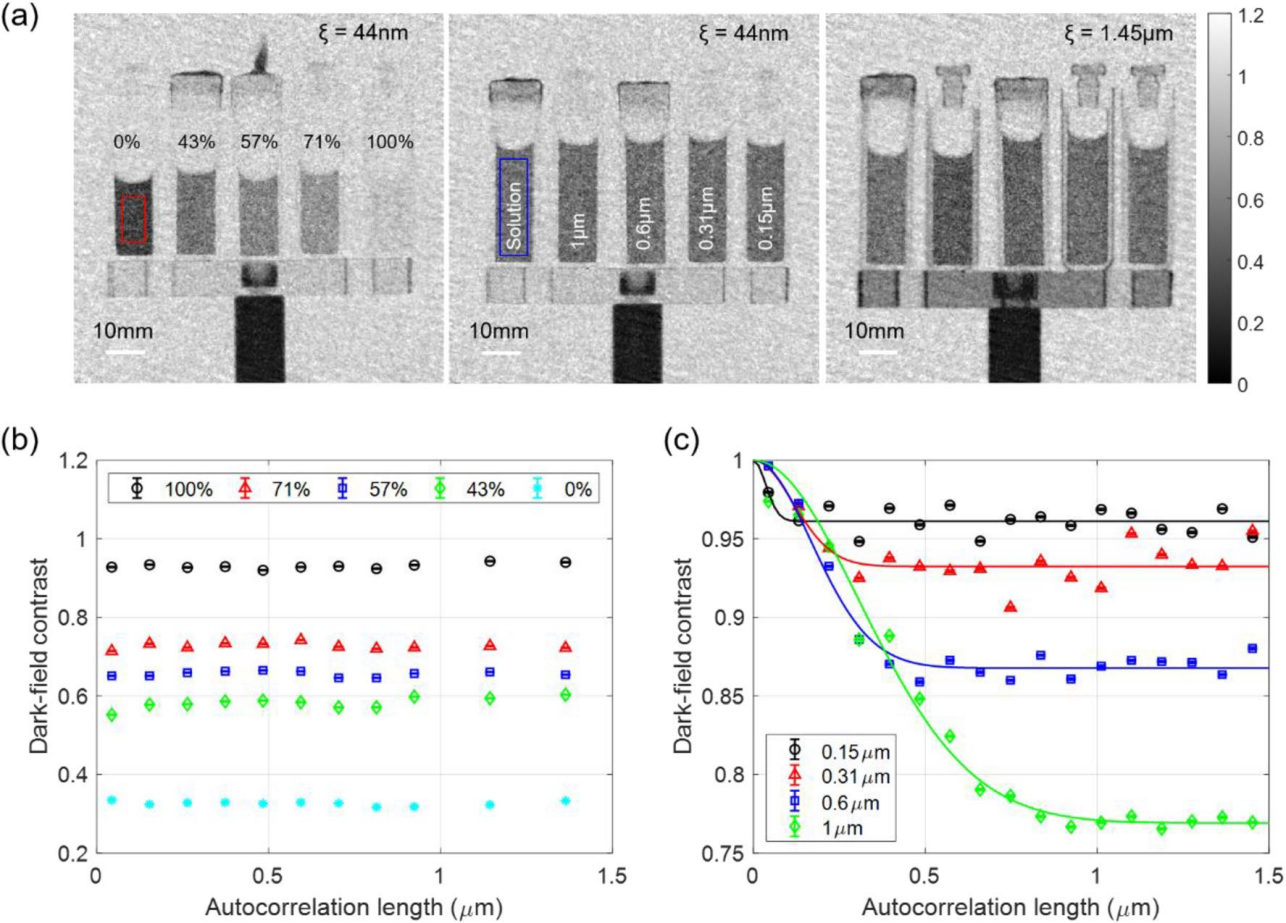
(**a**) Dark-field images of pure aqueous solutions with different D_2_O concentrations (left) and diluted polystyrene particle solutions (middle and right) before the correction for incoherent scattering from the aqueous solution. Dark-field contrast according to the ACLs of (**b**) pure aqueous solutions with different D_2_O concentrations and (**c**) diluted polystyrene particle solutions after the correction for the incoherent scattering from the aqueous solution. Other than D_2_O, the pure aqueous solutions only contain H_2_O. The dark-field contrast of diluted polystyrene particle solutions is corrected as dividing by the dark-field contast of the aqueous solution. The solid lines represent nonlinear least square fitting curves based on measured data.

The optically thick Gadox analyzer grating of the inverse nTLI results in reduced systematic uncertainties stemming from incoherent scattering as compared to the symmetric nTLI^23^. As shown in Fig. 3 in Ref.^23^, the dark-field contrast based on the ACL follows the inverse square law, which clearly represents the incoherent neutron scattering of H_2_O. As the ACL increases (i.e., distance between the sample and the detector increases), the dark-field contrast related to incoherent neutron scattering disappears because the intensity of the incoherently scattered neutrons is strongly reduced. The incoherent scattered neutrons generated by H_2_O at small ACLs (i.e., sample positions near the detector) are read by a detector over a Gadox structure with a low thickness, which reduces the visibility, resulting in corresponding poor measurement of dark-field contrast relatively. Therefore, the Gadox structure with a thickness of 150 μm (i.e., the height of analyzer grating in the inverse nTLI) is considered to have sufficient thickness to prevent incoherent scattered neutrons from affecting the dark-field contrast compared to the Gadox structure with a thickness of 20 μm (i.e., the height of analyzer grating in the symmetric nTLI in Ref.^23^). This is equivalent to the neutron grids that remove scattered neutrons in conventional attenuation imaging^32–34^. Therefore, as shown in Fig. 5(b), the dark-field contrast of a pure aqueous solutions exhibits a relatively constant value according to the ACL. This is the dark-field contrast caused by coherent scattering in each aqueous solution shown in Fig. 5(a) (left).

### Quantitative dark-field imaging of diluted polystyrene particle solutions

The results of the quantitative dark-field imaging of diluted polystyrene particle solutions are presented in Fig. 5. The dark-field images of diluted polystyrene particle solution at different autocorrelation lengths before the correction for incoherent scattering from the aqueous solution are shown in Fig. 5(a) (middle and right), and the dark-field contrast along the full measured range of autocorrelation length after the correction for incoherent scattering from the aqueous solution is shown in Fig. 5(c). The dark-field contrast of the diluted polystyrene particle solutions is corrected for the aqueous solutions by simply dividing the value by the dark-field contrast of the pure aqueous solution to extract the scattering from the polystyrene particles only. The error bars are calculated based on the error propagations for the standard errors of each ROI (red box, 100 × 200 pixels) in the pure aqueous solutions and each ROI (blue box, 120 × 400 pixels) in the diluted polystyrene particle solutions. All ROIs are processed by the median filter (3 × 3 pixels window) to remove non-statistical noise and measure a representative dark-field contrast. The solid lines are fitting curves that represent the isolated sphere model calculated via nonlinear square fitting with a priori knowledge regarding the neutron scattering length densities of the polystyrene and pure aqueous solutions. The fitting results exhibit good agreement with the measured data for each diluted polystyrene particle solution and the reduced chi-square of 12, 27, 10, and 24 for sphere sizes from 0.15 μm to 1 μm, indicating a good fit for the isolated sphere model. The structural parameters of sphere size and concentration extracted through the fitting process are listed in Table 2. The extracted structural parameters also agree well with the nominal structural parameters when accounting for errors in measurement and sample preparation, except for the polystyrene particle of 0.31 μm. The extracted concentration of polystyrene particles of 0.31 μm from the fitting process is lower than the nominal concentration by a factor 2, however, supported by the relatively larger sphere size of the fitting result than the nominal size.

### Quantitative dark-field imaging of silicon comb structures

**Figure 6:**
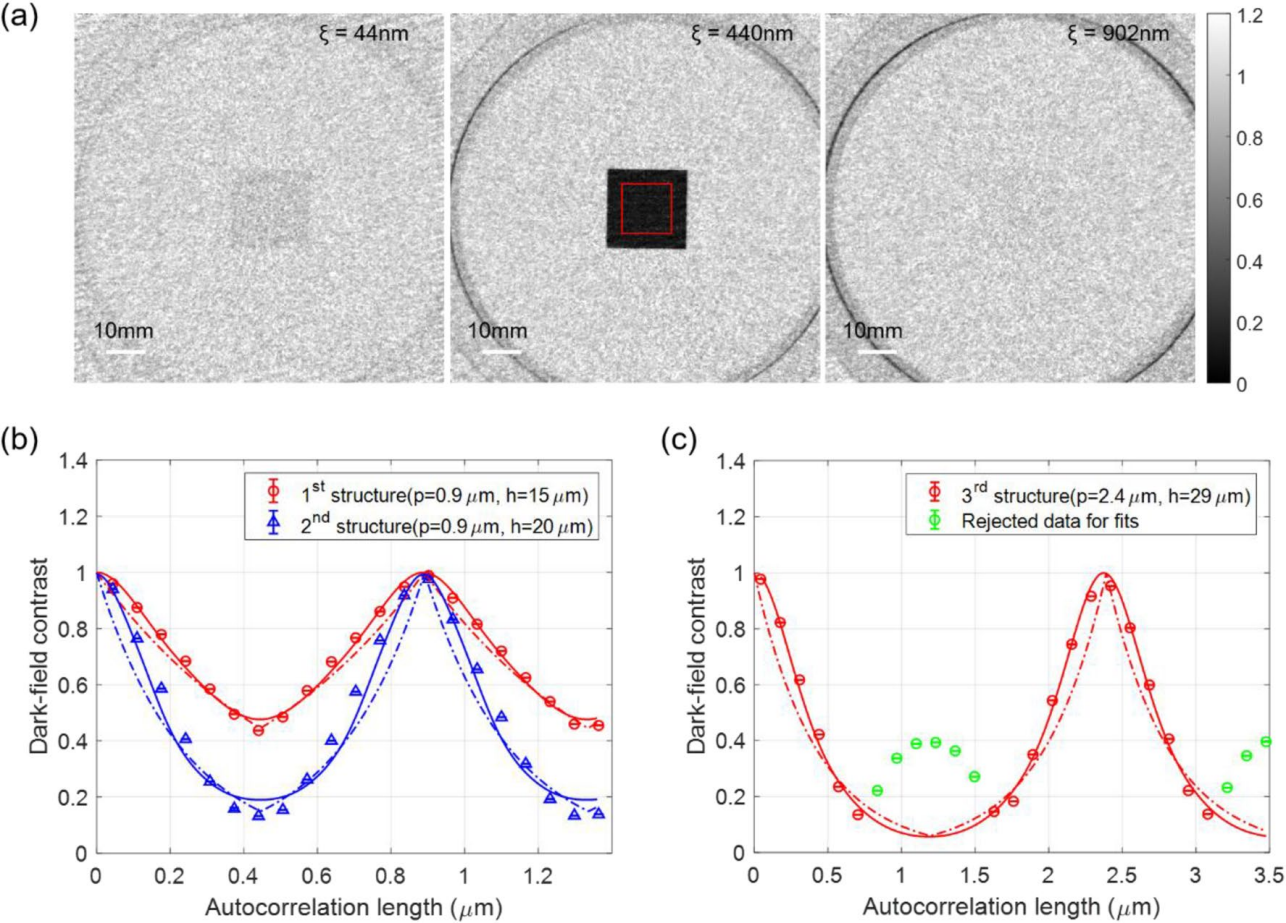
(**a**) Dark-field images of a silicon comb structure with a period of 0.9 μm and height of 15 μm (first structure). Dark-field contrast according to the ACLs of the (**b**) first and second structures, and (**c**) third structure. Dash-dotted lines represent nonlinear least square fitting curves using rectangular shape and solid lines represent nonlinear least square fitting curves using trapezoidal and specific shapes based on measured data. In (**c**), the green points have occurred by the imaginary part, which is generated due to the phase shift of the third structure, and they have been rejected for the fitting process.

Figure 6(a) presents dark-field images of the silicon comb structure (G_3_), which has a period of 0.9 μm and height of 15 μm (first structure). The silicon comb structure exhibits different dark-field contrast values at different ACLs, whereas the unfabricated area of the silicon wafer exhibits a constant dark-field contrast for all measured ACLs.

The results of quantitative dark-field imaging of the silicon comb structure for each period and height are presented in Fig. 6(b) and (c). Figure 6(b) represents the first and second structures and Fig. 6(c) represents the third structure. The presented dark-field contrast values are the average values of the ROI (red box, 250 × 250 pixels) and the standard errors of the ROI are represented by the error bars. As expected, the dark-field contrast attains its maximum at $\xi =0$ and $\xi =p$ and its minimum at the half-period of the structure $\xi =p/2$.

For the dark-field measurement for the first and second structures according to autocorrelation lengths, deviations from the fits obtained by nonlinear least square fitting of the periodic bar model shown as dash-dotted lines. As discussed above in Fig. 2, the SEMs of the silicon comb structures show clear deviation from a rectangular shape due to the etching process^35^. For example, the deformation in the ridge of the silicon comb structure can occur in the top region under the mask material. In this regarding, it is considered that the actual shape (trapezoidal or specific shapes) of a silicon comb structure has a specific ridge-to-trench ratio, as shown in Fig. 2. The solid lines in Fig. 6 correspond to nonlinear least square fits using the oriented periodic bar model of actual shapes shown in Fig. 3. The deviation between model and measured data decreases compared to that of the rectangular shape model. The improved fit of the actual shapes can be represented by the reduced-chi square of 70, 1600, and 360 for from first structure to third structure compared to the reduced-chi of 330, 6500, and 2900 for the rectangular structure. The structural parameters of the period, height, and duty cycle of silicon comb structure extracted through the fitting process are listed in Table 3 and the extracted structural parameters also agree well with the nominal structural parameters and the parameters measured by the SEM.

**Table 3:** Structural parameters of silicon comb structure based on the oriented periodic bars of actual shapes.

	Nominal			Experimental (fitted)		
	Period (μm)	Height (μm)	Duty cycle	Period (μm)	Height (μm)	Duty cycle
1st structure	0.9	15	0.5	0.89 ± 0.01	14.45 ± 0.10	0.50 ± 0.00
2nd structure	0.9	20	0.5	0.89 ± 0.01	20.01 ± 0.49	0.51 ± 0.00
3rd structure	2.4	29	0.5	2.37 ± 0.01	28.45 ± 0.12	0.50 ± 0.00

Note that errors represent confidence bounds on parameters of the confidence level of 95%.

For the third structure, as shown in Fig. 6(c), the measured dark-field contrast differs from the model of the oriented periodic bars, particularly at the autocorrelation length of a half of the period. The measured dark-field contrast at $\xi =p/2$ is not the minimum value, but rather a secondary peak. One thing to consider here to understand the secondary peak of third structure is that the silicon comb structure (G_3_) works as an additional phase grating in the grating interferometer. As with the principle of the grating interferometer, the silicon comb structure in the interferometer induces a corresponding phase shift as to the neutron wavelength, resulting a specific interference pattern over the distance, namely the projection (i.e., the intensity profile) of the silicon comb structure on the detector plane (or the analyzer plane). For example, in the near-field interference regime, the silicon comb structure of π-phase shift produces the projection of the structure with a half of period at the fractional Talbot distance, which can be expressed by the combination of even-order and odd-order peaks of the projections of the structure with a period. The odd-order peaks are dominant to the real-space shape of the silicon comb structure, but the even-order peaks are dominant to the real-space projection of the silicon comb structure which is not the real-space shape of the structure, and which is resulted from the interference, thus called imaginary part. As the height of the silicon comb structure deceases so the phase shift decreases, the even-order peaks of the projection become relatively weaker compared to the odd-order peaks of the projection, so that at certain phase shift the total projection of the silicon comb structure resembles the actual real-space shape of the structure, with the even-order peaks of the projection vanishing and only the odd-order peaks of the projection remaining. The details of the interference pattern are in Supplementary Materials.

In the experiment, the silicon phase grating G_2_ of 34.39 μm height induces a π-phase shift for a neutron wavelength of 4.4 Å, from which the first, second, and third silicon comb structures (G_3_) of the sample of 15 μm, 20 μm, and 29 μm height of silicon provide 0.44π-, 0.58π-, and 0.87π-phase shifts, respectively. Through the interference pattern simulation (in Supplementary Materials), the even-order peaks of the projection generated from the first and second structures are rarely existed, whereas the third structure has the strong even-order peaks of the projection, resulting in an imaginary part of the silicon comb structure corresponding to a half period $\xi =p/2$. Finally, in the quantitative dark-field imaging that measures the silicon comb structure above a certain phase shift, the projection of the structure relating to the imaginary part is carefully considered in the analysis process. Hence, the green data points in Fig. 6(c) represent the imaginary part of the third structure and they have been rejected for the fitting process. The dash-dotted and solid lines represent fitting curves that represent the oriented periodic bars of rectangular and trapezoidal shapes respectively via nonlinear least square fitting with a priori knowledge regarding the neutron scattering length densities of the silicon. The fitting result of trapezoidal shapes shows reasonable agreement with the measured data, and the extracted structural parameters from the fitting process listed in Table 3 also agree well with the nominal structural parameters. These results resonate with results of neutron sub-micrometre tomography from scattering data^36^ of silicon phase gratings.

## Conclusions

In this paper, an inverse nTLI was introduced for quantitative dark-field imaging. For neutron imaging, the inverse nTLI improves visibility based on its large absorption grating height, resulting in a maximum visibility of 52%. Additionally, the wide period of the analyzer grating significantly extends the ACL range, which begins at low tens of nanometers, and the minimum ACL of 44 nm was demonstrated. Quantitative dark-field imaging was demonstrated using diluted polystyrene particle solutions and silicon comb structures. The microstructure of the diluted polystyrene particle solutions, which had a size below 1 μm, was analyzed using an isolated sphere model. The systematic error due to incoherent neutron scattering at shorter ACLs was improved by the strong neutron-absorbing capabilities of the thicker analyzer grating. The silicon comb structure was analyzed using the oriented periodic bar model with the actual shapes, and the structural characteristics of the period, height, and duty cycle were estimated. In the analysis, it was observed that the imaginary parts should be considered for the periodic phase object which of the projection was different from the real shape of object. In summary, the inverse nTLI enables quantitative dark-field imaging to access a sufficiently extended ACL range that is inaccessible for the conventional setup and symmetric setup. The success of the inverse nTLI will facilitate the application of dark-field imaging to various material studies that have traditionally been conducted using SANS, thereby facilitating the acquisition of both microscopic structural features and macroscopic spatial images.

## Supplementary Information

Supplementary Information.
