# Supplementary material for: Analysis of a silicon comb structure using an inverse Talbot–Lau neutron grating interferometer
Source: Sci Rep. 2022 Mar 3;12:3461. doi: 10.1038/s41598-022-06409-y (PMC8894421; doi:10.1038/s41598-022-06409-y)
Supplement: Supplementary file 1 — Supplementary Information. [file 41598_2022_6409_MOESM1_ESM.docx]

**Analysis of a silicon comb structure using an inverse Talbot-Lau neutron grating interferometer**

Youngju Kim^1,2,3^, Daeseung Kim^1^, Daniel S. Hussey^3^, Jongyul Kim^4^, Mona Mirzaei^5,6,7^, Dmitry A. Pushin^5,6^, Charles W. Clark^8^, Seung Wook Lee^1*^

^1^School of Mechanical Engineering, Pusan National University, Busan, Republic of Korea.

^2^Department of Chemistry and Biochemistry, University of Maryland, College Park, MD, USA.

^3^Neutron Physics Group, National Institute of Standards and Technology, Gaithersburg, MD, USA.

^4^Neutron Science Division, Korea Atomic Energy Research Institute, Daejeon, Republic of Korea.

^5^Institute of Quantum Computing, University of Waterloo, Waterloo, ON, Canada.

^6^Department of Physics, University of Waterloo, Waterloo, ON, Canada.

^7^Science Systems and Applications, Inc., Lanham, Maryland, USA

^8^Joint Quantum Institute, National Institute of Standards and Technology and the University of Maryland, Gaithersburg, MD, USA.

^*^Electronic mail: [seunglee@pusan.ac.kr](mailto:seunglee@pusan.ac.kr)

**Supplementary materials**

In order to examine the projection of the silicon comb structure (i.e., a binary Ronchi grating of silicon, G_3_), a numerical simulation of the interference pattern in the grating interferometer was performed. The wave is determined by a path integral of all neutrons originating from the source and passing through the grating, which is expressed by applying the Fresnel-Kirchhoff diffraction integral, and the integral is sequentially conducted until the interference pattern of final detection area (red box in Figure 1) is obtained. The schematics of grating interferometer is shown in Figure 1 in the manuscript.

The wave originated from the neutron source is expressed in front of the source grating (G_1_) as

|  | $E_{0}\left( x_{0},x_{1},L_{0} \right)=e^{ik\sqrt{{L_{0}}^{2}+\left( x_{1}-x_{0} \right)^{2}}}$, | (S1.1) |
| --- | --- | --- |

where $k={2\pi}/\lambda$ is the wave vector, $x_{0}$ is the position of the neutron source, $x_{1}$ is the position of the G_1_, and $L_{0}$ is the distance between the neutron source and G_1_. The wave passed through G_1_ is expressed by

|  | $E_{1}\left( x_{2},L_{1} \right)=\int{e^{ik\sqrt{{L_{1}}^{2}+\left( x_{2}-x_{1} \right)^{2}}}t_{1}\left( x_{1} \right)e}^{ik\sqrt{{L_{0}}^{2}+\left( x_{1}-x_{0} \right)^{2}}}dx_{1} ,$ | (S1.2) |
| --- | --- | --- |

where $t_{1}$ is the transfer function of G_1_ with a period $p_{1}$, which is expressed as a Fourier series by

|  | $t_{1}\left( x_{1} \right)=\sum_{n} A_{n}e^{i2\pi n\frac{x_{1}}{p_{1}}} .$ | (S1.3) |
| --- | --- | --- |

The Fourier constant $A_{n}$ of G_1_ for the neutron absorption grating is given by

|  | $A_{n}=\left\{ \begin{aligned} T_{1}\mathrm{sinc}\left( f_{1}n \right)+\left( 1-T_{1} \right)\mathrm{sinc}\left( f_{1}n \right) n\neq0 \\ T_{1}+\left( 1-T_{1} \right)f_{1} n=0 \end{aligned} , \right.$ | (S1.4) |
| --- | --- | --- |

where $\mathrm{sinc}$ is the normalized sinc function, $T_{1}$ is the transmission rate, and $f_{1}$ is the duty cycle of G_1_ which is a ratio of grating material width to grating period.

The wave passed through the phase grating (G_2_) on the $x_{2}$-axis is expressed by

|  | $E_{2}\left( x_{3},L_{2} \right)=\int E_{1}\left( x_{2},L_{1} \right)t_{2}\left( x_{2} \right)e^{ik\sqrt{{L_{2}}^{2}+\left( x_{3}-x_{2} \right)^{2}}}dx_{2} ,$ | (S1.5) |
| --- | --- | --- |

where $t_{2}$ is the transfer function of G_2_ with a period $p_{2}$ and is expressed by

|  | $t_{2}\left( x_{2} \right)=\sum_{m} B_{m}e^{i2\pi m\frac{x_{2}}{p_{2}}} ,$ | (S1.6) |
| --- | --- | --- |

with Fourier constant $B_{m}$ of G_2_ for neutron phase grating of $\Phi$ phase shift and duty cycle $f_{2}$,

|  | $B_{m}=\left\{ \begin{aligned} \mathrm{sinc}\left( m \right)+\left( e^{i\Phi}-1 \right)f_{2}\mathrm{sinc}\left( f_{2}m \right) m\neq0 \\ 1+\left( e^{i\Phi}-1 \right)f_{2} m=0 \end{aligned} \right. .$ | (S1.7) |
| --- | --- | --- |

The wave after the silicon comb structure (G_3_) on the $x_{3}$-axis, which is at final detection area, is expressed by

|  | $E_{3}\left( x_{4},L_{3} \right)=\int E_{2}\left( x_{3},L_{2} \right)t_{3}\left( x_{3} \right)e^{ik\sqrt{{L_{3}}^{2}+\left( x_{4}-x_{3} \right)^{2}}}dx_{3}$ | (S1.8) |
| --- | --- | --- |

where $t_{3}$ is the transfer function of G_3_ with a period $p_{3}$ and is expressed by

|  | $t_{3}\left( x_{3} \right)=\sum_{w} C_{w}e^{i2\pi w\frac{x_{3}}{p_{3}}} ,$ | (S1.9) |
| --- | --- | --- |

with Fourier constant $C_{w}$ of G_3_ for neutron phase grating (Eq. (S1.7)).

Under the paraxial (small-angle) approximation^[[1]](#footnote-1)^, Eq. (S1.8) can be discretized by using the Gaussian intergal^[[2]](#footnote-2)^:

|  | $E_{3}\left( x_{4},L_{3} \right)=\sum_{w} C_{w}\sum_{m} B_{m}\sum_{n} A_{n}\sqrt{\frac{\pi}{-a}}\sqrt{\frac{\pi}{-\alpha}}\sqrt{\frac{\pi}{-q}}e^{-\frac{r^{2}}{4q}+s} ,$ | (S1.10) |
| --- | --- | --- |

where

|  | $a=\frac{ik}{2L_{0}}+\frac{ik}{2L_{1}}$ | (S1.11) |
| --- | --- | --- |
|  | $\alpha=\frac{ik}{2L_{1}}-\frac{ikL_{0}}{2L_{1}\left( L_{0}+L_{1} \right)}+\frac{ik}{2L_{2}}$ | (S1.12) |
|  | $q=\frac{ik}{2L_{2}}-\frac{ik\left( L_{0}+L_{1} \right)}{2L_{2}\left( L_{0}+L_{1}+L_{2} \right)}+\frac{ik}{2L_{3}}$ | (S1.13) |
|  | $r=-\frac{ikx_{0}}{\left( L_{0}+L_{1}+L_{2} \right)}+\frac{i2\pi nL_{0}}{p_{1}\left( L_{0}+L_{1}+L_{2} \right)}+\frac{i2\pi m\left( L_{0}+L_{1} \right)}{p_{2}\left( L_{0}+L_{1}+L_{2} \right)}+\frac{i2\pi w}{p_{3}}-\frac{ikx_{4}}{L_{3}}$ | (S1.14) |
|  | $s=ikL_{0}+ikL_{1}+ikL_{2}+ikL_{3}+\frac{i2\pi nL_{1}x_{0}}{\left( L_{0}+L_{1} \right)p_{1}}+\frac{i2\pi mL_{2}x_{0}}{\left( L_{0}+L_{1}+L_{2} \right)p_{2}}$ | (S1.15) |
|  | $+\frac{i2\pi nL_{0}L_{2}x_{0}}{\left( L_{0}+L_{1} \right)\left( L_{0}+L_{1}+L_{2} \right)}+\frac{ik{x_{0}}^{2}}{2L_{0}}-\frac{ikL_{1}{x_{0}}^{2}}{{2L}_{0}\left( L_{0}+L_{1} \right)}-\frac{ikL_{2}{x_{0}}^{2}}{2\left( L_{0}+L_{1} \right)\left( L_{0}+L_{1}+L_{2} \right)}$ |  |
|  | $-\frac{i2\pi^{2}n^{2}L_{0}L_{1}}{k{p_{1}}^{2}\left( L_{0}+L_{1} \right)}-\frac{i2\pi^{2}n^{2}{L_{0}}^{2}L_{2}}{k{p_{1}}^{2}\left( L_{0}+L_{1} \right)\left( L_{0}+L_{1}+L_{2} \right)}$ |  |
|  | $-\frac{i4\pi^{2}nmL_{0}L_{2}}{kp_{1}p_{2}\left( L_{0}+L_{1}+L_{2} \right)}-\frac{i2\pi^{2}m^{2}L_{2}\left( L_{0}+L_{1} \right)}{k{p_{2}}^{2}\left( L_{0}+L_{1}+L_{2} \right)}+\frac{ik{x_{4}}^{2}}{2L_{3}} .$ |  |

Finally, the intensity of wave function represents the interference pattern in the detection area on the $x_{4}$-axis is determined by

|  | $I_{3}\left( x_{4},L_{3} \right)=\left\vert E_{3}\left( x_{4},L_{3} \right) \right\vert^{2} .$ | (S1.16) |
| --- | --- | --- |

The projection of G_3_ is the line profile of $I_{3}$ which is generated onto the analyzer grating (G_4_) surface on the $x_{4}$-aixs, hence the detection plane is $x_{4}$ and the path intergal analysis have not included G_4_.

Figure S1 shows the projection (i.e., the intensity of profile) of G_3_ as to the phase shift $\Phi$ and the position of G_3_. The position (the distance between G_3_ and the detection plane, L_3_) is related to the autocorrelation length (ACL, $\xi$), which equals to quarters of the period (p_3_) of silicon comb structure.

For the π-phase shift (Figure S1(d)), the projection has the intensity profile of silicon comb structure (G_3_) with the half of period, ${p_{3}}/2$. That is, the projection is a combination of odd-order and even-order peaks of the projection of G_3_ with period, $p_{3}$. As the height of G_3_ decreases so the phase shift becomes smaller (Figure S1(a)-(c)), the even-order peaks of the projection of G_3_ becomes relatively weak compared to the odd-order peaks, and the even-order peaks can be vanished from a certain phase shift, finally producing the intensity profile of G_3_ with a period, $p_{3}$. It represents the odd-order peaks are dominant to the real-space shape of G_3_, but the even-order peaks are dominant to the real-space projection of G_3_ which is not the real-space shape of G_3_ and which is resulted from the interference, thus called the imaginary part. The projection of silicon comb structure (G_3_) with 0.87π-phase shift (the silicon structure of 30 μm in height at the neutron wavelength of 4.4 Å) has the strong odd-order peaks of the projection and the relatively weak but non-negligible even-order peaks of the projection for all positions as shown in Figure S1(c).

It explains the secondary peak in the dark-field contrasts of the third silicon comb structure shown in Figure 6(c) in the manuscript. The dark-field contrast along the ACL reveals the projected real-space correlation function, which is the autocorrelation of the real-space projection of the silicon comb structure. Hence, the correlation of the real-space shape of the structure must be considered rather than the real-space projection of the structure, especially if the real-space structure and the real-space projection are different such as a periodic phase object (i.e., silicon comb structure). That is, the dark-field contrast along the ACL only for the actual real-space shape of the structure corresponds to the odd-order peaks of the projection should be selected in the silicon comb structure analysis. The projection of silicon comb structures with 0.44π-phase shift and 0.57π-phase shift (the height of 15 μm and 20 μm, respectively) has the strong odd-order peaks of the projection and the relatively weak even-order peaks of the projection at the position L_3_($\xi$ =p_3_) as shown in Figure S1(a) and (b). Nevertheless, unlike the silicon comb structure with large phase-shift, the dark-field contrast is minimal at the ACL of half-grating period shown in Figure 6(b) in manuscript is due to the relatively negligible even-order peaks of the projection with a bias value. At other positions of L­_3_($\xi$ =1/4p_3_) and L­_3_($\xi$ =3/4p_3_), the projection corresponding to exactly a period of the silicon comb structure is created.


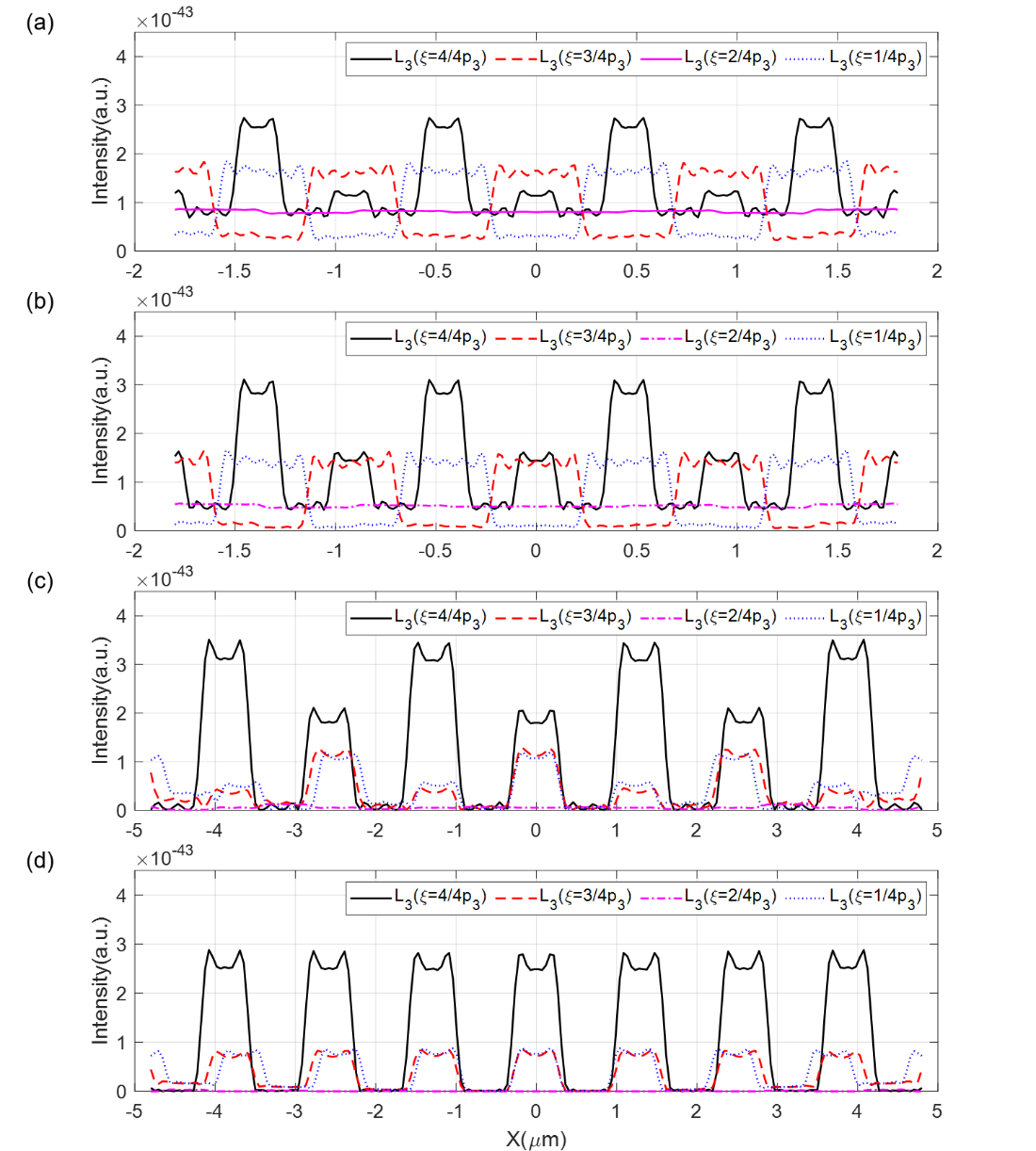


Figure S1. The various projections of G_3_ with different phase shifts and positions of G_3_. The position (L_3_) corresponds to the ACL, $\boldsymbol{\xi}$, which equals to quarters of period (p_3_) of the silicon comb structure. (a) Silicon comb structure with 0.9 μm period and 15 μm height (resulting 0.44π-phase shift at the neutron wavelength of 4.4 Å), (b) Silicon comb structure with 0.9 μm period and 20 μm height (resulting 0.58π-phase shift at the neutron wavelength of 4.4 Å), (c) Silicon comb structure with 2.4 μm period and 29 μm height (resulting 0.87π-phase shift at the neutron wavelength of 4.4 Å), (d) Silicon comb structure with 2.4 μm period and 34.39 μm height (resulting π-phase shift at the neutron wavelength of 4.4 Å).

1. $\sqrt{{L_{0}}^{2}+\left( x_{1}-x_{0} \right)^{2}}\cong L_{1}+\left( \frac{1}{2L_{1}} \right)\left( {x_{1}}^{2}-2x_{1}x_{0}+{x_{0}}^{2} \right)$ [↑](#footnote-ref-1)
2. $\int e^{-ax^{2}+bx+c}dx=\sqrt{\frac{\pi}{a}}e^{\frac{b^{2}}{4a}+c}$ [↑](#footnote-ref-2)
